# A post-labeling method for multiplexed and multicolored genotyping analysis of SSR, indel and SNP markers in single tube with bar-coded split tag (BStag)

**DOI:** 10.1186/1756-0500-4-161

**Published:** 2011-05-26

**Authors:** Tokurou Shimizu, Kanako Yano

**Affiliations:** 1 Okitsu Citrus Research Station, National Institute of Fruit Tree Science. 485-6 Okitsu Nakacho, Shimizu-ku, Shizuoka 424-0292, Japan

## Abstract

**Background:**

Genotyping analysis using capillary DNA sequencing with fluorescently labeled primer pairs obtained by polymerase chain reaction (PCR) is widely used, but is expensive. The post-PCR labeling method using fluorescently labeled short oligonucleotides and nested PCR of the amplified product obtained from unlabeled primer pairs is a simple and inexpensive alternative. However, previously reported protocols often produced spurious peaks or inconsistent amplification under multiplexed analysis as a result of simultaneous progress of both the amplification and labeling reactions and local homology of the attached tag sequence.

**Results:**

A set of 16 bp-long oligonucleotide sequences termed bar-coded split tag (BStag), comprising a common basal region, a three-nucleotide 'bar-code' sequence, and a mismatched nucleotide at the middle position were designed for selective post-PCR labeling. The BStag was attached at the 5' end of the forward primer of interest. The melting temperature of the BStag was low enough to separate the labeling reaction from initial PCR amplification, and each sequence was minimally divergent but maintained maximum selectivity. Post-PCR labeling of the amplified product was achieved by extending for three cycles at a lower annealing temperature after the conventional amplification program with the appropriate fluorescently labeled BStag primer. No amplification was confirmed with BStag primers for 12 plant species. The electropherogram of the labeled product obtained using this method was consistent with that of prelabeled primer, except for their apparent size.

**Conclusions:**

BStag enabled multiplexed post-PCR labeling of simple sequence repeat or insertion/deletion markers with different dyes in a single tube. BStag in conjunction with locus specific oligo and allele specific oligo was also useful for single nucleotide polymorphism analysis. The labeling protocol was simple and no additional operation was required. Single-tube multiplexed post-PCR labeling is useful for a wide variety of genotyping studies with maximal flexibility and minimal costs.

## Backgrounds

Various types of DNA markers including simple sequence repeat (SSR), insertion/deletion (indel), or single nucleotide polymorphism (SNP) have been developed and used in a wide variety of genotyping studies with a fluorescent capillary DNA sequencer [1]. Although rapid developments in whole genome sequencing technology or array-based assay systems have enabled large-scale discovery and genotyping of polymorphisms, genotyping analysis using a fluorescent capillary DNA sequencer remains important for small- to mid-scale evaluation due to its ease of use, accuracy, and moderate cost. Fluorescently labeled primers are used in these studies, although their synthesis costs are expensive, and the entire performance of each analysis is strictly dependent on the total number of DNA markers used.

As such, there has been a concerted effort to reduce the total costs by developing a post-PCR labeling technique that avoids use of fluorescently prelabeled primers. For example, Iwahana et al. demonstrated incorporation of fluorescently labeled deoxyribotrinucleotide for a PCR amplified product by terminal exchange activity of a Klenow fragment [2]. Inazuka et al. improved this original method by attaching a three nucleotide tail at the 5' end to one of the primer pairs to incorporate a specific dye nucleotide [3]. That protocol was used for incorporation of two different fluorescent dyes for each strand of PCR product, and was successfully used in Single-Stranded Conformational Polymorphism (SSCP) analysis [4]. However, these methods still required a time-consuming additional labeling step after PCR amplification.

An alternative postlabeling technique using a tagged primer for labeling was also proposed [5-7]. The unlabeled, but tagged, forward primer was mixed with the corresponding reverse primer and a fluorescently labeled primer then provided for the PCR amplification. The nucleotide sequence of interest was amplified with an unlabeled target specific primer pair during the PCR reaction, but was also amplified with a fluorescently labeled primer from the tagged sequence simultaneously by nested PCR. This is a simple and inexpensive method for post-PCR labeling due to the availability a variety of fluorescent dyes compatible with modern fluorescent capillary sequencing, and has a highly flexibility with respect to the nucleotide sequence of the tag, multiplexed analysis, and dye-swapping. Multiplexed labeling of up to four SSR markers with single dye-labeled tag primer was initially reported [6], and a simplified protocol for single dye labeling was proposed [7]. This protocol was further improved to allow multiplexed postlabeling for up to two markers in single tube [8]. Multiplexed analysis by mixing three amplified products that were preliminary labeled individually with three fluorescent dyes with different tag primers was also reported [9].

We initially applied these protocols for multiplexed post-PCR labeling of several primer pairs that were intended to be labeled with different fluorescent dyes in single tube. In our preliminary use of these methods, however, we often encountered spurious peaks not observed when fluorescently labeled primer pairs were used. The peaks were prominent under multiplexed analyses that contained several markers in a single tube. We also found inconsistencies in the apparent intensities of the amplified products depending on the combination of tagged sequence and plant samples used. Simultaneous progress for both the amplification and labeling reactions was proposed to alter the amount of fluorescently labeled product due to the close annealing temperature between the attached tag sequence and the sequence-specific primer. Separating the amplification of the unlabeled target and the fluorescent labeling would be sufficient to eliminate this problem, although this further complicates the reaction protocol. Local homology of the tagged sequence against the adjacent region of the primer pair was also proposed to affect amplification efficiency, resulting in a discrepancy of the apparent intensity of the amplified product. As such, we deduced that a set of nucleotide sequences with a low annealing temperature and sufficient specificity, but with minimum diversity, would allow for stable multiplexed post-PCR labeling analysis. Thus, we designed a set of oligonucleotide sequences termed bar-coded split tag (BStag) suitable for selective post-PCR labeling of SSR, indel, and SNP markers. Application and availability of BStag for post-PCR labeling of SSR, indel, and SNP markers with different fluorescent dyes in single tube are described below.

## Results

### Design and initial evaluation of BStag sequence

**Figure 1:**
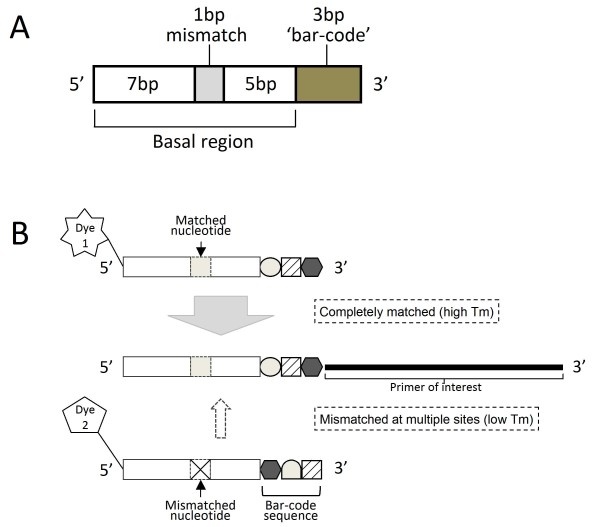
**Structure and principle of bar-coded split tag**. A. Schematic structure of bar-coded-split tag. This tag was a 16 bp oligo nucleotide consisting of a 13 bp basal region and a 3 bp bar-code region. A mismatch was introduced at the 8th nucleotide from the 5' end for the combination of a similar bar-code sequence to disturb annealing to the complementary sequence and to enhance selectivity. B. Principle of selective labeling with BStag. A complementary strand of PCR product amplified with an oligonucleotide primer harboring a 16 bp-long BStag sequence at its 5' end (center) anneals to the fluorescently labeled BStag primer with same sequence (upper panel). A fluorescently labeled BStag primer with similar sequence should not anneal to the complementary strand due to breakage of the annealing by the mismatched nucleotide at the bar-code sequence and the basal region (lower panel).

BStag sequences were designed to have low melting temperatures allowing isolation of the labeling reaction from initial PCR amplification with minimum divergence, while maintaining maximum selectivity. The BStag sequences consisted of three parts: basal region, 'bar-code' sequence, and a mismatched nucleotide at the middle position (Figure 1A). The basal region was a conserved nucleotide sequence among a set of BStag sequences that was developed from the nucleotide sequence of a 12 mer random amplified polymorphic DNA (RAPD) marker known to give no amplified product upon wide variety of PCR conditions and citrus cultivars. The conserved nucleotide sequence at the basal region was expected to minimize the influence of polymorphic nucleotides that could hybridize within a region for an attached primer upon a variety of DNA templates. The 'bar-code' sequence was a three nucleotide-long short sequence at the 3' end of BStag that provided sequence-specific annealing of an individual BStag primer for the corresponding tag sequence attached to the sequence-specific primer. The 'bar-code' sequence was selected from a combination of four nucleotides that were intended to retain a G or C residue at their 3' terminal for GC-clamped landing. The BStag sequences were distinguished from each other by the bar-coded sequences; however, three nucleotide bar-code sequences were insufficient to suppress misamplification between similar sequences even under a considerably higher annealing temperature. We also introduced a nucleotide mismatch at the middle position of the BStag sequence to split the basal region into two discontinuous regions. Accordingly, any two BStag sequences should include a discrepancy at the mismatched nucleotide at the middle position and the tag sequence at their 3' end (Figure 1B). Split sequences (seven nucleotides) were too short to bind any similar sequence on the complementary strand under usual PCR conditions, and effectively destabilized hybridization among BStag sequences with a similar bar-code sequence. A perfectly matched sequence was long enough to bind the specific sequence under conventional PCR conditions, and had sufficient specificity to discriminate a specific primer from the others used.

We initially designed a total of 20 candidate BStag sequences and confirmed no homology against nucleotide sequences of expressed sequence tags or whole genome shotgun of citrus in public DNA databases with BLASTN search. BStags were evaluated for their ability to produce no product at annealing temperatures between 52-56°C with citrus genomic DNA for template. Selected BStag sequences were again confirmed to give no amplified product between all combinations of two of the primers, and then six BStag sequences were selected (Table 1). These selected sequences were also confirmed to give no amplified product with template DNA from apple, Japanese pear, Japanese chestnut, cherry, soybean, cucumber, eggplant, tomato, watermelon, red pepper, and spinach (data not shown).

**Table 1:** Nucleotide sequences of BStag primers.

Tag name	Sequence	Tm (°C)
F9GAC	5' CTAGTATCAGGACGAC 3'	51.3
F9GTC	5' CTAGTATGAGGACGTC 3'	51.3
F9TAC	5' CTAGTATCAGGACTAC 3'	47.2
F9GCC	5' CTAGTATTAGGACGCC 3'	51.7
F9CCG	5' CTAGTATTAGGACCCG 3'	50.8
F9AGG	5' CTAGTATTAGGACAGG 3'	47.5

Nucleotide sequences of six BStags. One of these tag sequences was attached to the 5' end of a forward primer of a sequence-specific primer. The double underlined three nucleotides represent a 'bar-code' sequence. The single underlined nucleotide corresponds to the mismatched nucleotide in Figure 1A. Estimated melting temperatures (Tm) were obtained with PerlPrimer version 3[13].

### Evaluation of post-PCR labeling for a single DNA marker

Four SSR primer sets that were initially designed for prelabeled primers from citrus EST sequences were used for evaluation (Table 1). Their annealing temperatures were at least 6°C higher than the BStag primers. One of the BStag sequences was attached to the 5' end of the forward primer of the four citrus SSR markers. The concentrations of unlabeled primers in reaction mixture were decreased to that used in prelabeled analyses to suppress unexpected amplification (Additional file 1, Figure S1). The PCR cycle at the initial stage was further extended by several cycles for the program of the prelabeled primers to compensate for the reduced product amplification resulting from decreased primer concentration. Part of the amplified product was labeled with fluorescently labeled BStag primer during the initial PCR stage, although the amount of labeled product was insignificant as the annealing temperature of BStag was lower than the sequence-specific primer at this stage. As the resulting concentration of amplified product was strictly dependent on the amount of tagged forward primer, the majority of the tagged primer was used by the end of amplification due to the reduced primer concentrations. Consequently, a considerable amount of the amplified DNA fragment that was synthesized from the reverse primer would be single stranded, as described by Schuelke [7]. The second stage of the reaction was performed at a lower temperature than in the initial stage. Fluorescently-labeled BStag primer bound the single stranded amplified DNA, and then synthesized another strand that retained fluorescent dye at the terminal.

**Figure 2:**
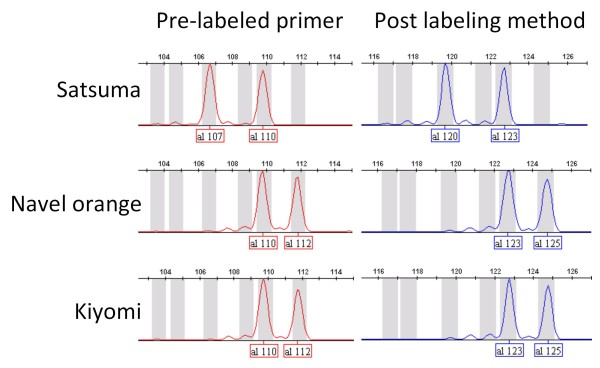
**Comparison of electropherogram for SSR marker analysis with two different labeling methods**. Electropherograms of SSR08B25 markers for Satsuma mandarin (*C. unshiu *Marc.), sweet orange (*C. sinensis *Osbeck), and 'Kiyomi' tangor (hybrid between Satsuma mandarin and sweet orange) obtained using a prelabeled primer pair with PET (left) and post-PCR labeling with 6-FAM (right).

**Figure 3:**
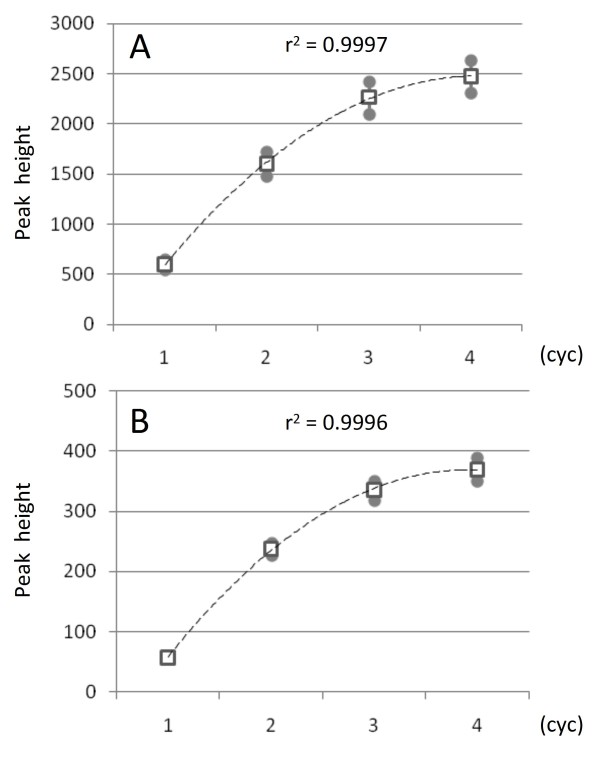
**Effect of repetitive post-PCR labeling reaction upon labeling efficiency**. The amount of fluorescently labeled product by post-PCR labeling with SSR08A04 (A) and SSR08B25 (B) markers were compared from one to four cycles. Peak intensities of 16 citrus accessions were averaged. Empty squares and vertical lines ending with a circle indicate average and standard errors, individually. Hatched line represents the second-order polynomial fitted curve, and equations in the graph represent the correlation coefficient.

Labeled amplified products obtained by post-PCR labeling gave identical electropherograms, but the apparent sizes of the postlabeled products were increased by approximately 13-17 bp compared with those of prelabeled products (Figure 2). The differences of apparent size of the amplified product obtained with the prelabeled primer versus post-PCR labeling were due to the attached fluorescent dye. The concentration of labeled product at the post-PCR labeling stage increased proportionally up to three cycles, and then reached a plateau at the fourth cycle (Figure 3). Peak intensities of postlabeled products were half to one-third of those obtained with prelabeled product due to the reduced amount of the tagged primer. Extending the labeling step slightly increased the amount of labeled product, although nonspecific spurious product could also appear occasionally. A mismatched combination of fluorescently labeled BStag primer and tag-labeled sequence-specific primer gave no detectable peaks in 96 different DNA samples. We evaluated a total of 201 primer sets for SSR markers with BStag and then confirmed fluorescently labeled distinct peaks for 196 primers. Peak intensities of the amplified products obtained with the failed five tagged primers were significantly lower than those of the other primers because of insufficient amplification during the initial PCR cycle. Comparison of genotypes obtained from a wide variety of citrus varieties using post-PCR labeling analysis was consistent with those obtained using the prelabeled primer, except for their size. Genetic analysis on a segregation population confirmed consistent inheritance for both genotypes as demonstrated in Figure 2. Application of BStag for genotyping of indel markers also demonstrated results consistent with those obtained using primer-labeled genotyping (data not shown).

### Application for multiplexed labeling in a single tube

**Figure 4:**
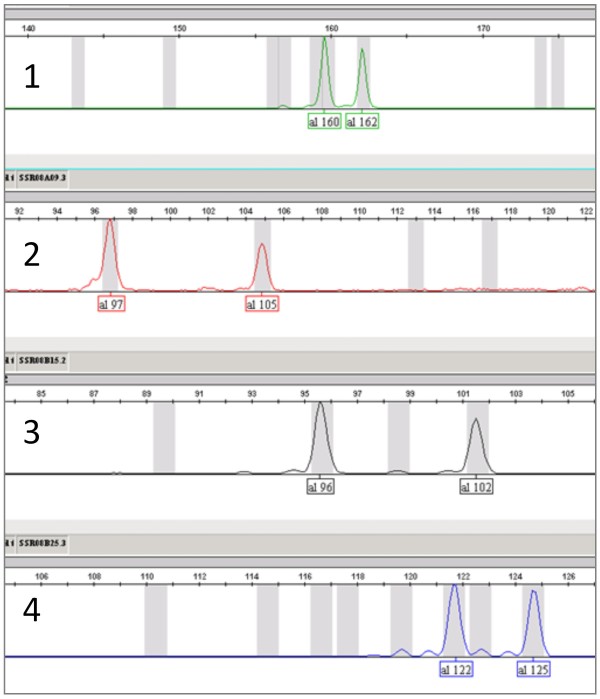
**Electropherogram of four SSR markers obtained by multiplexed post PCR labeling in a single tube**. Genotyping of four SSR markers listed in Table 2 successfully gave individual peaks labeled with different fluorescent dyes in a single tube. 1) SSR08A04/F9GCC + VIC (green). 2) SSR08A09/F9GTC + PET (red). 3) SSR08B15/F9GAC + NED (black). 4) SSR08B25/F9TAC + 6-FAM (blue). Numbers under each peak represent estimated fragment size. These electropherograms were obtained for Binkitsu (*C. platymamma *hort. ex Tanaka).

A multiplexed post-PCR labeling of four SSR markers in a single PCR reaction mixture confirmed exclusive dye labeling and no cross-labeling (Figure 4, Table 2). Analysis was the same that used for single markers. Genotypes obtained using multiplexed analysis were consistent with those obtained using single marker analysis. In multiplexed analysis, the amount of the amplified product is decreased several fold compared with that of single marker analysis due to competition for dNTPs and Taq enzyme among the amplifying products following combination of sequence-specific primers and corresponding fluorescently labeled BStag primers into the single PCR mixture. Thus, we directly mixed an aliquot of PCR reaction mixture with formamide without modifying the PCR program, and then denatured for fragment analysis. Both the multiplexed analysis and elimination of the dilution step drastically reduced plastic waste, time to operation, and total cost of analysis.

**Table 2:** Nucleotide sequences of SSR markers for post-PCR labeling.

Marker (DDBJ ID)	Motif		BStag/dye	Sequence (5' to 3')
SSR08A04	(aaac)n	Forward	F9GCC/VIC	CTAGTATTAGGACGCCCAAACACTTATTCGGGATCAG
				
(DY293653)		Reverse		gtttcttAATGCCATTATCAAACCGCC

SSR08A09	(cagg)n	Forward	F9GTC/PET	CTAGTATGAGGACGTCCATCATTGCATCAGCATCAC
				
(CX663959)		Reverse		gtttcttAGTTCTACATCATAACCTGCC

SSR08B15	(acc)n	Forward	G9GAC/NED	CTAGTATCAGGACGACCCCATACTTGAAACCAAACC
				
(DY292389)		Reverse		gtttcttAGCAGCATGACTTAATCCA

SSR08B25	(tta)n	Forward	F9TAC/6-FAM	CTAGTATCAGGACTACCGTTGACTCTCAAGAGATCTG
				
(DY289996)		Reverse		gtttcttCTTCACAGTCCGCAGCATTA

Each marker was provided for genotyping with a BStag primer labeled with fluorescent dye. The nucleotide sequences of the attached BStag primer in the forward primers are underlined. The nucleotide sequences underlined and small capitals represent the 'pig-tail' sequence [14].

### Application for SNP typing

**Figure 5:**
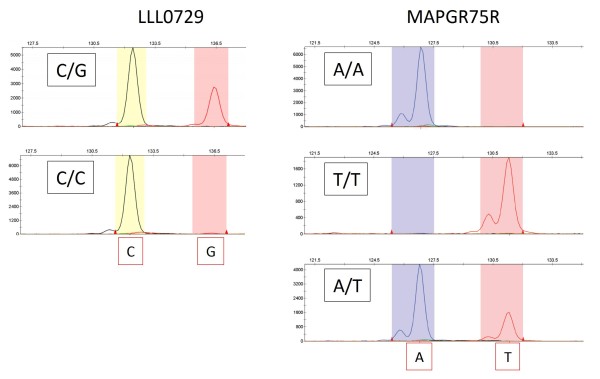
**An application of post-PCR labeling with BStag for SNP markers**. Two SSR markers listed in Table 3 demonstrated successful labeling of each allele with different colors using BStag. A marker LLL0729 demonstrated segregation of C/G alleles for two different citrus hybrids (C/G: a hybrid of *C. kunip *× *C. kinokuni*; C/C: a hybrid of 'Lee' × *C. kinokuni*) labeled with NED (allele C) and PET (allele G). Another marker MAPGR75R also demonstrated segregation of A/T alleles for three citrus accessions: A/A for 'Hayasaki' (*C. grandis *'Mato' × *C. grandis *'Hirado'), T/T for a hybrid of *C. kunip *× *C. kinokuni*, and A/T for Satsuma mandarin.

We applied PCR amplification in combination with locus specific oligo (LSO) and allele specific oligo (ASO) to identify single nucleotide polymorphisms. Nucleotide sequences for ASO and LSO were designed from citrus EST for SNP analysis. The LSO primer was a common primer for these ASO primers, and was designed from a conserved nucleotide sequence closely adjacent to the site of the SNP. Each ASO sequence was designed to keep the sequence identical, but harbor a polymorphic SNP nucleotide at the 3' end. Locally GC-rich nucleotide sequence or a contiguous stretch of G or C for more than five residues were excluded for ASO primer design, as they were rather stable and difficult for allele specific amplification under usual PCR conditions. Because a polymorphic nucleotide at the 3' terminal of ASO was usually insufficient to discriminate a different allele, we designed ASO sequences by introducing mismatched nucleotides within the ASO sequence at -1 to -6 position from the 3' end by replacing C or G nucleotide to A or T to destabilize mishybridization between different ASOs. Different BStag sequences were attached at the 5' terminal of each ASO primer for post-PCR labeling. The tagged ASO primers also differed by 1 bp in length to guarantee definitive separation of each allele. As a result, SNP genotyping in conjunction with post-PCR labeling gave distinct peaks for individual alleles that were labeled with different fluorescent dyes (Figure 5, Table 3). The SNP genotypes obtained with BStag analysis were confirmed to be identical to those obtained by sequence analysis for 16 citrus varieties. Each SNP allele was easily distinguished from others by differences in color and size on the capillary DNA sequencer. The apparent fragment size was also affected by the fluorescent dye attached, which facilitated the reliable genotyping by eliminating overlap.

**Table 3:** Nucleotide sequences of SNP markers attached with BStag sequences for post-PCR labeling.

Marker (DDBJ ID)	SNP	Type	BStag/dye	Sequence (5' to 3')
LLL0729 (DC891931)	(C/G)	LSO	-	gtttcttGAAGTAGAAGGATGATAATCAC
		ASO/C	F9GAC/NED	CTAGTATCAGGACGACATGGACACAACTCAAGTt**C**
		ASO/G	F9GTC/PET	CTAGTATGAGGACGTCAATGGACACAACTCAAGTt**G**

MAPGR75R (DC893380)	(A/T)	LSO	-	gtttcttGGTTGGTATATCTACGAGAC
		ASO/T	F9GTC/PET	CTAGTATGAGGACGTCTATCCGATTGAGCCTTTc**T**
		ASO/A	F9TAC/6-FAM	CTAGTATCAGGACTACATCCGATTGAGCCTTTc**A**

The underlined nucleotide sequences in lower case represent the 'pig-tail' sequence in LSO. The underlined nucleotide sequences in upper case represent the BStag attached to ASO. The nucleotide in bold type at the 3' end of each ASO corresponds to a polymorphic nucleotide, and the adjacent underlined lower case character represents the mismatched nucleotide. A set of LSO and ASO primers were used for PCR reaction with corresponding fluorescently labeled BStag primers.

## Discussion

The designed BStag was short enough to be attached as a tag for a sequence-specific primer of interest, and was appropriate for both single and multiplexed post-PCR labeling analysis. Post-PCR labeling was achieved by simply extending the initial amplification for several cycles longer than those used for the prelabeled primer sets, followed by three cycles at a decreased annealing temperature. No laborious and time-consuming additional operations were required for post-PCR labeling, and the PCR program for the prelabeled primer was usually applicable for post-PCR labeling of BStag without further optimization. Consequently, this allowed fast and simple analysis with minimum changes of protocol used for prelabeled primer set. The low BStag annealing temperature effectively prevented appearance of spurious peaks caused by random priming of template DNA during target amplification. The adjacent nucleotide sequence of the sequence-specific primer corresponding to the region of the attached BStag sequence can affect amplification efficiency, but this was kept minimal as 12 out of 16 nucleotides were conserved among a set of BStag sequences. The total cost of the genotyping by post-PCR labeling with BStag was as low as one-tenth to one-twentieth of that for prelabeled primers.

Estimated genotypes obtained by the BStag method were identical to those obtained with prelabeled primers except for their product size. We designed six BStag primers and were able to perform multiplex analysis for up to six different markers in a single tube if they had a different labeled dye or had a different size of amplified product. Even in case where two of DNA markers in same tube were different for BStag, but their product size and fluorescent dye overlapped, the duplicated dye could be swapped for a different color by replacing the same BStag primer labeled with a different fluorescent dye.

When applying this method for the analysis of any primer pairs, the following considerations are recommended: 1) the primer sequence should be designed to produce the target amplified product without extra bands, 2) the PCR program for amplification should be optimized before post-PCR labeling, 3) fluorescent dye set attached to BStag should be carefully selected to minimize interference among them by overlap of their emission spectrum, and 4) the specificity of SNP analysis simply depends on primer design of LSO and ASO. Many types of primer design for SNP analysis have been reported. The amplified product length polymorphism (APLP) method [10,11] would be simple and useful for design of a set of primer sequences for SNP genotyping in conjunction with BStag.

## Conclusion

We demonstrated that BStag was useful for genotyping with SSR and indel markers, with less cost and minimal modification of the PCR program used for prelabeled primers. Genotyping with SNP markers in combination with mismatched ASO primers also extended the application of BStag. These features provided versatile multiplexed post-PCR labeling for the combination of several DNA markers with different fluorescent dyes in a single reaction tube. A sufficient number of BStag primers allowed for easy replacement of fluorescent dyes, which is a problem in conventional multiplexed analysis. Although we confirmed that the BStag primer itself gave no amplified product on twelve different plants, it may give spurious amplification in other organisms. However, the simple principle of BStag allows easy development of new BStag primer sets with different nucleotide sequences. In conclusion, post PCR labeling with BStag can be used in a wide variety of genotyping studies with maximal flexibilities and minimal cost.

## Methods

### Genotyping analysis

Unlabeled DNA primers were purchased from Integrated DNA Technologies, Inc. (Coralville, IA, USA). Template DNA used in this study was extracted from mature leaves of citrus using the CTAB method [12]. PCR reactions for prelabeled primers labeled with 6-FAM, VIC, NED, or PET at their 5' end (Life Technologies) were performed in a 10 μL reaction mixture that consisted of 2.5 ng of template DNA, 1X KAPA 2G buffer A, 200 nM dNTP, 0.5 mM MgCl_2_, 4 pmoles of forward and reverse primers, and 0.1 U KAPA 2G Fast DNA polymerase (KAPA Biosystems Inc., Woburn, MA, USA). PCR amplification for the prelabeled primer was performed in a PE 9700 cycler (Life Technologies) at 94°C for 3 min of initial denaturation following by 28-30 cycles of amplification at 94°C for 20 s, 54-62°C for 30 s, and final extension at 72°C for 10 min. Labeled samples were diluted 30-100 fold with distilled water, and then a 0.9 μL aliquot was mixed with 0.18 μL of GeneScan™ 600 LIZ size marker (Life Technologies) and adjusted to 20 μL with deionized formamide. Fragment size analysis was performed with an ABI PRISM 3130xl genetic analyzer (Life Technologies) and a POP-7 polymer with a 36 cm-long glass capillary using a standard fragment analysis program. Peak detection, size estimation, and allele calling were performed using GeneMapper software (ver.4, Life Technologies). Primer concentrations in the reaction mixture for post PCR labeling were decreased to 2 pmole for the reverse primer and 0.5 pmole for both the tagged forward primer and the fluorescently labeled BStag primer. Target-specific amplification and post PCR labeling was performed with two-staged PCR cycles. The amplified product was labeled by an additional three cycles at 94°C for 20 s, 49°C for 10 s, and 72°C for 5 s before final extension.

### Multiplexed genotyping analysis

The composition of the reaction mixture and the PCR program for multiplexed analysis was the same as those for single markers. After the PCR reaction, 0.9 μL of the mixture was directly mixed with deionized formamide then applied for genotyping, as for single marker analysis.

### SNP genotyping analysis

The PCR mixture for SNP genotyping with separation and detection was the same as for SSR marker analysis, but consisted of 2 pmole of LSO primer and 0.5 pmole of ASO primers with corresponding BStag primers labeled with fluorescent dye. Following analysis procedure was same for single marker analysis.

## Competing interests

The authors declare that they have no competing interests.

## Authors' contributions

TS developed the principle of BStag analysis, designed their sequences, performed most experiments, and prepared the manuscript. KY contributed to evaluate the majority of SNP analysis. All authors read and approved the final manuscript.

## Supplementary Material

Additional file 1 **Supplementary Figure S1: Influence of excess fluorescently labeled BStag primer on production of spurious peaks**. Increasing the amount of F9GCC + VIC (green) primer with SSR08A04 from 0.5 (1) to 1.0 (2), 1.5 (3) and 2.0 (4) pmole per reaction mixture causes amplification of a nonspecific peak (red arrow). Other experimental conditions are equal to those described in Figure 4. Click here for file

## References

[B1] Nguyen HT Xiaolei Wu Khalid Meksem GK Molecular marker systems for genetic mapping The handbook of plant genome mapping 2005 Weinheim: WILLEY-VCH GmbH & Co 23 52

[B2] Iwahana H Adzuma K Takahashi Y Katashima R Yoshimoto K Itakura M Multiple fluorescence-based PCR-SSCP analysis with postlabeling PCR Methods Appl 1995 4 5 275 282 7580914 10.1101/gr.4.5.275

[B3] Inazuka M Tahira T Hayashi K One-tube post-PCR fluorescent labeling of DNA fragments Genome Research 1996 6 6 551 557 10.1101/gr.6.6.551 8828044

[B4] Inazuka M Wenz HM Sakabe M Tahira T Hayashi K A Streamlined Mutation Detection System: Multicolor Post-PCR Fluorescence Labeling and Single-Strand Conformational Polymorphism Analysis by Capillary Electrophoresis Genome Research 1997 7 11 1094 1103 9371745 10.1101/gr.7.11.1094 PMC310684

[B5] Neilan BA Wilton AN Jacobs D A universal procedure for primer labelling of amplicons Nucleic Acids Res 1997 25 14 2938 2939 10.1093/nar/25.14.2938 9207046 PMC146818

[B6] Oetting WS Lee HK Flanders DJ Wiesner GL Sellers TA King RA Linkage Analysis with Multiplexed Short Tandem Repeat Polymorphisms Using Infrared Fluorescence and M13 Tailed Primers Genomics 1995 30 3 450 458 10.1006/geno.1995.1264 8825630

[B7] Schuelke M An economic method for the fluorescent labeling of PCR fragments Nat Biotechnol 2000 18 2 233 234 10.1038/72708 10657137

[B8] Guo DC Milewicz DM Methodology for using a universal primer to label amplified DNA segments for molecular analysis Biotechnol Lett 2003 25 24 2079 2083 14969412 10.1023/b:bile.0000007075.24434.5e

[B9] Missiaggia A Grattapaglia D Plant microsatellite genotyping with 4-color fluorescent detection using multiple-tailed primers Genet Mol Res 2006 5 1 72 78 16755499

[B10] Watanabe G Umetsu K Suzuki T Determination of the HUMTH01 alleles by the APLP method Int J Legal Med 1999 112 2 134 135 10.1007/s004140050216 10048673

[B11] Watanabe G Umetsu K Yuasa I Suzuki T Amplified product length polymorphism (APLP): a novel strategy for genotyping the ABO blood group Hum Genet 1997 99 1 34 37 9003490 10.1007/s004390050306

[B12] Murray MG Thompson WF Rapid isolation of high molecular weight plant DNA Nucleic Acids Res 1980 8 19 4321 4325 10.1093/nar/8.19.4321 7433111 PMC324241

[B13] Marshall OJ PerlPrimer: cross-platform, graphical primer design for standard, bisulphite and real-time PCR Bioinformatics 2004 20 15 2471 2472 10.1093/bioinformatics/bth254 15073005

[B14] Brownstein MJ Carpten JD Smith JR Modulation of non-templated nucleotide addition by Taq DNA polymerase: primer modifications that facilitate genotyping Biotechniques 1996 20 6 1004 1006 1008-1010. 8780871 10.2144/96206st01

