# Supplementary figures and images for: A post-labeling method for multiplexed and multicolored genotyping analysis of SSR, indel and SNP markers in single tube with bar-coded split tag (BStag)
Source: BMC Res Notes. 2011 May 26;4:161. doi: 10.1186/1756-0500-4-161 (PMC3126724; doi:10.1186/1756-0500-4-161)

## Slide 1
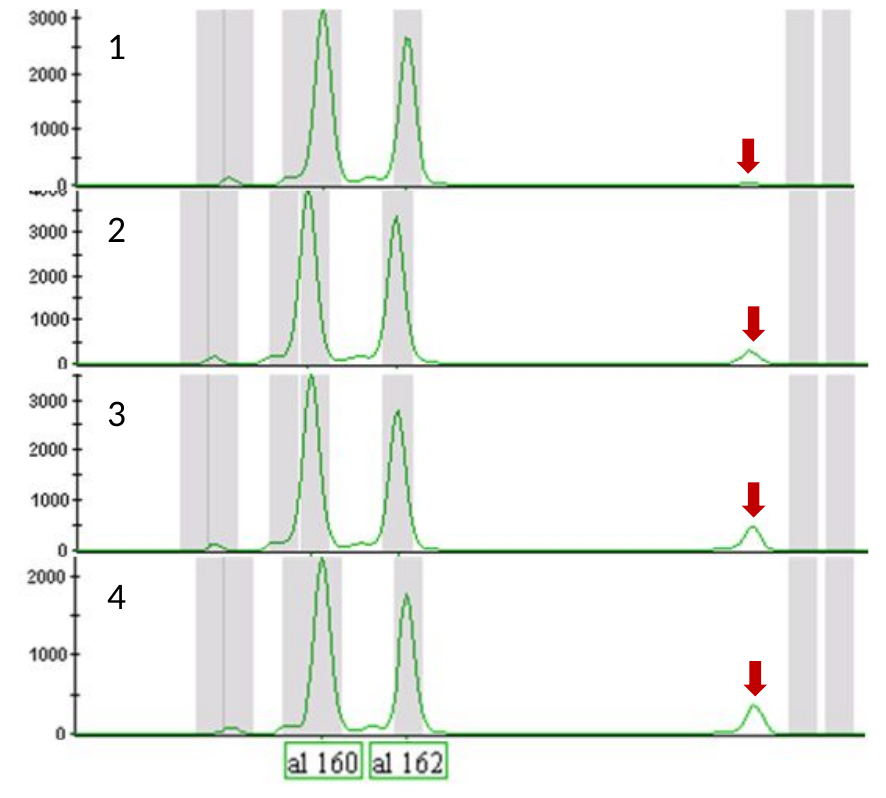

1
2
3
4

Supplement: Additional file 1 — Supplementary Figure S1: Influence of excess fluorescently labeled BStag primer on production of spurious peaks. Increasing the amount of F9GCC + VIC (green) primer with SSR08A04 from 0.5 (1) to 1.0 (2), 1.5 (3) and 2.0 (4) pmole per reaction mixture causes amplification of a nonspecific peak (red arrow). Other experimental conditions are equal to those described in Figure 4. [file 1756-0500-4-161-S1.PPT]
